# Infectious *v*. Non-Infectious Convalescents

**Published:** 1887-03-05

**Authors:** 


### The Editor's Letter-Box.

[Our Correspondents are reminded that prolixity is a great bar to publication, and that brevity of style and conciseness of statement greatly facilitate early publication.]

### INFECTIOUS *v.* NON-INFECTIOUS CONVALESCENTS.

"Sister Clara" writes: "A friend and myself, both hospital-trained nurses (daughters of medical men), are desirous of opening a house at some seaside or bracing inland place, for the reception of invalids of the upper classes recovering from illness, requiring change and attention. We are anxious to know if such a place is in request, or, if a house for the reception of such, recovering from *infectious* diseases, would be more successful. If medical men, or others, would give any information on the subject, through the columns of your invaluable paper, we shall feel greatly indebted and grateful. We should be glad to know which coast would be the most advisable."

